# Invariance-based causal prediction to identify the direct causes of suicidal behavior

**DOI:** 10.3389/fpsyt.2022.1008496

**Published:** 2022-11-14

**Authors:** Austin V. Goddard, Yu Xiang, Craig J. Bryan

**Affiliations:** ^1^Department of Electrical and Computer Engineering, The University of Utah, Salt Lake City, UT, United States; ^2^Department of Psychiatry and Behavioral Health, The Ohio State University, Columbus, OH, United States

**Keywords:** suicide, causal prediction, military, primary care (MeSH), invariance

## Abstract

Despite decades of research, the direct causes of suicide remain unknown. Some researchers have proposed that suicide is sufficiently complex that no single variable or set of variables can be determined causal. The invariance-based causal prediction (ICP) is a contemporary data analytic method developed to identify the direct causal relationships, but the method has not yet been applied to suicide. In this study, we used ICP to identify the variables that were most directly related to the emergence of suicidal behavior in a prospective sample of 2,744 primary care patients. Fifty-eight (2.1%) participants reported suicidal behavior during the following year. Of 18 predictors tested, shame was most likely to be directly causal only under the least restrictive conditions. No single variable or set of variables was identified. Results support the indeterminacy hypothesis that suicide is caused by many combinations of factors, none of which are necessary for suicide to occur.

## Introduction

Suicide has consistently ranked as a top 10 cause of death in the U.S. for many years (1). From 1999 to 2018, the U.S. suicide rate increased by over 30% (1) before declining slightly in 2019 (2). Suicide rates may be underestimated, however, due to some deaths potentially being misclassified as accidents (3, 4). Researchers have identified many suicide risk and protective factors but most are only weakly correlated with suicidal behavior (5). The “direct causes” of suicide therefore remain unknown. Controlled experiments provide the strongest method for establishing causal relationships but can be costly and impractical. Correlation-based and regression-based approaches are much easier to conduct but causation typically cannot be inferred because these approaches cannot account for the effects missing variables that may influence both the predictor and the outcome, resulting in spurious findings. Researchers have therefore sought to develop alternative methods for causal inference that balance pragmatism with inferential confidence.

One increasingly popular approach is machine learning, a set of computationally intensive methods that can be especially useful when analyzing potentially complicated relationships among many predictor variables and an outcome, especially non-linear and interactive relationships (6) that characterize the assumed causes of suicidal behavior (7, 8). Despite the rapid expansion of machine learning methods in suicide research, findings to date typically fail to replicate across samples (5). Furthermore, algorithms based on different combinations of risk and protective factors predict suicide with similar accuracy (5, 9, 10), leading some researchers to conclude that no single variable or set of variables directly causes suicide (11). Although machine learning provides an especially powerful tool for examining factors associated with suicidal behavior, causation typically cannot be inferred (6).

An alternative method for identifying the direct causes of key outcomes is invariance-based causal prediction (ICP) (12). Like machine learning methods, ICP analyzes complicated relationships among many predictor variables and an outcome. Unlike machine learning methods, however, ICP provides a mechanism for inferring causality based on the assumption that the conditional distribution of the outcome given its direct causes will not change when other variables are modified or manipulated. In other words, a variable can be considered directly causal if its relationship with suicidal behavior remains constant across settings and circumstances but a variable is unlikely to be directly causal if its relationship with suicidal behavior changes across settings and circumstances. ICP also provides a mechanism for evaluating the indeterminacy hypothesis: if ICP analyses fail to identify one or more causal variables, indeterminacy would be supported.

To our knowledge, ICP has not been used to guide or inform suicide prevention research, although preliminary evidence from other medical disciplines supports the method's utility for identifying causal mechanisms underlying other important health conditions like breast cancer and hypertension (13, 14). ICP could therefore identify key mechanisms and targets underlying the emergence of suicidal behavior. The primary aim of this study was to use ICP to identify variables that are most directly related to the emergence of suicidal behavior. Owing to the nature of the analyses employed, we did not specify an a priori hypothesis regarding any single variable or set of variables that we expected to be causal predictors. To achieve this objective, we conducted a secondary analysis of data collected as part of the PRImary care Screening Methods (PRISM) study, a longitudinal cohort study aimed at identifying optimal methods for suicide prevention screening in primary care medical settings (15).

## Methods

### Participants and procedures

Participants were 2744 primary care patients recruited from 6 military primary care clinics across the U.S. from July 2015 to August 2018 by researchers who were positioned in the clinic waiting rooms. Patients were invited to complete a survey battery before or after a routine clinic visit. Interested patients provided consent to participate, after which they completed a self-report survey battery *via* laptop or computer tablet. Upon completion, patients were offered a small token of appreciation for their time (e.g., military challenge coin, t-shirt, $5 gift card for a coffee shop). Researchers contacted patients 6 and 12 months postbaseline to assess the incidence of suicidal behaviors since enrollment using the Self-Injurious Thoughts and Behaviors Interview (SITBI; 16), described below. Patients were eligible for inclusion if they were at least 18 years of age, eligible to receive medical care from the Department of Defense, and able to understand the English language. the only exclusion criterion was the inability to provide informed consent due to an acute medical or psychiatric condition that diminished mental status (e.g., acute intoxication, altered consciousness, psychosis). These eligibility criteria were selected to maximize the generalizability of findings to the population of interest: primary care patients. Additional details about study procedures can be found in Bryan et al. (15).

### Outcome variable: Suicidal behavior

Suicidal behavior was assessed at baseline and follow-up using the SITBI (16), an assessment instrument designed to distinguish and characterize different forms of suicidal ideation (*Have you ever had thoughts of killing yourself?*), suicidal behavior (*Have you ever made an actual attempt to kill yourself in which you had at least some intent to die?*), and non-suicidal self-injury (*Have you ever actually purposely hurt yourself without wanting to die?*). During follow-up, participants were asked these same questions but the assessment timeframe was changed from “ever” to “within the past 6 months” or “within the past 12 months.” The SITBI's reliability and validity are established (16).

### Predictor variables

Eighteen empirically supported risk and protective factors were selected as candidate causal variables. The variables listed below represent common symptoms and constructs that are used both clinically and in research as indicators of risk factors for suicide. Even though the list is not exhaustive, the variables selected were based on a combination of theory and empirical findings. For example, entrapment is central to the integrated motivational-volitional theory of suicide (17), fearlessness about death is central to the interpersonal psychological theory of suicide (18), maladaptive beliefs are central to the fluid vulnerability theory of suicide (8), and depression and suicidal ideation are well-established suicide risk factors whereas social support is a well-established protective factor (5). The instruments used to assess these variables are described below with internal consistency estimates (i.e., Cronbach's alpha) derived from the present sample.

#### Guilt, shame, and inward hostility

The Differential Emotions Scale-IV (DES-IV) (19) is a self-report scale designed to measure 12 distinct emotional states. Each emotional state is assessed with 3 items each. Items are rated using a 5-point scale, with higher scores indicating greater frequency of experiencing each emotion. Three subscales were administered in this study: guilt, shame, and inward hostility. Cronbach's alphas in the current sample were, respectively, 0.92, 0.89, and 0.92.

#### Entrapment

The Entrapment Scale (ES) (20) is a 16-item self-report scale designed to measure external entrapment (e.g., “I am in a situation I feel trapped in”) and internal entrapment (e.g., “I want to get away from myself”). Ten items are used to assess internal entrapment and 6 items are used to assess external entrapment. Items are rated using a 5-point scale, with higher scores indicating more severe levels of entrapment. Cronbach's alphas in the current sample were, respectively, 0.95 and 0.94.

#### Social support

The shortened version of the Interpersonal Support Evaluation List (ISEL-12) (21) is a 12-item self-report scale designed to measure three components of perceived social support: appraisal (i.e., availability of advice or guidance from others), belonging (i.e., feeling empathy and acceptance from others), and tangible (i.e., material help or assistance). Each subscale is assessed with 4 items. Items are rated using a 5-point scale, with higher scores indicating stronger perceptions of each type of support. Cronbach's alphas in the current sample were, respectively, 0.68, 0.72, and 0.57.

#### Positive and negative affect

The International Positive and Negative Affect Scale-Short Form (I-PANAS-SF) (22) is a 10-item self-report scale designed to measure positive (5 items) and negative affect (5 items). Items are rated using a 5-point scale, with higher scores indicating more intense positive or negative experience. Cronbach's alphas in current sample were, respectively, 0.86 and 0.88.

#### Post-traumatic stress symptoms

The Primary Care Posttraumatic Stress Disorder Checklist (PC-PTSD) (23) is a 4-item self-report scale designed to screen for a diagnosis of PTSD. The scale's items assess the presence or absence of PTSD symptoms within the past month using a yes/no response format. The Kuder-Richardson estimate in the current sample was 0.86.

#### Depression symptoms

The Patient Health Questionnaire Depression Subscale (PHQ-9) (24) is a 9-item self-report scale designed measure the 9 symptoms of a major depressive episode during the past 2 weeks. In this study, we omitted the suicide ideation item (item 9) to examine suicidal ideation as an independent predictor. Items are rated using a 4-point scale, with higher scores indicating greater frequency of each symptom. to assess depression severity. Cronbach's alpha for the full scale in the current sample was 0.90.

#### Suicidal ideation

The ninth item of the PHQ-9 assesses the frequency of “thoughts that you would be better off dead, or thoughts of hurting yourself in some way” within the past 2 weeks. The item is rated using a 4-point scale, with higher scores indicating more frequent suicidal ideation.

#### Maladaptive beliefs

The Suicide Cognitions Scale-Revised (SCS-R) (25) is a 16-item self-report scale designed to measure maladaptive suicidogenic beliefs and perceptions commonly reported by suicidal patients (e.g., “I can't stand this pain anymore” and “Nothing can help solve my problems”). Items are rated using a 5-point scale, with higher scores indicating greater identification with negative self-perceptions and worldviews. Cronbach's alpha in the current sample was 0.97.

#### Fearlessness about death

The Acquired Capability for Suicide Scale-Fearlessness About Death (ACSS-FAD) (26) is an 8-item self-report scale designed to assess perceived fear of death (e.g., “The fact that I am going to die does not affect me” and “I am very much afraid to die”). Items are rated using a 5-point scale, with higher scores indicating less fear of death (i.e., more fearlessness). Cronbach's alpha in the current sample was 0.71.

#### Reasons for living

The Brief Reasons for Living Inventory (BRFLI) (27) is a 14-item self-report scale that assesses adaptive beliefs and expectations for living. Items are rated using a 6-point rating scale, with higher scores indicating a stronger motive to remain alive or not attempt suicide. Cronbach's alpha in the current sample was 0.89.

#### Sleep disturbance

The Insomnia Severity Index (ISI) (28) is a 7-item self-report scale that assesses the severity of sleep disturbance and the impact of sleep disruption on one's life. Items are rated using a 5-point scale, with higher scores indicating greater subjective sleep disturbance. Cronbach's alpha in the current sample was 0.93.

### Environmental variables: Demographics and prior suicide risk

ICP assumes we have knowledge from multiple environments. While environments can be created using controlled experimental designs, a benefit of ICP is that the conditions under which the environments were created can be unknown. Thus, we have considerable freedom in choosing environments, albeit with some constraints. First, a variable can only be a valid environment if it is neither a causal descendent nor direct causal parent of the outcome variable (29). Demographic and historical variables are valid environments because they are conditions that existed prior to data collection and did not develop because of suicidal behavior. Second, environmental variables must also be correlated with the outcome. If the outcome does not change across environments then the environmental conditions are not, from the perspective of the outcome, sufficiently distinct. The environmental variables used in this analysis included self-reported race (White vs. non-White), prior suicidal ideation (yes vs. no), prior suicide attempt (yes vs. no), prior non-suicidal self-injury (yes vs. no), age (≤23 years vs. 24+ years), and self-reported gender (male vs. female).

### Data analyses

We used the invariant causal prediction (ICP) framework described by Peters et al. (12), which assumes the conditional distribution of the outcome given its direct causal predictors is invariant to all interventions on variables other than the outcome (i.e., the environmental variables). This invariance, however, will not hold if the conditioning does not consist purely of direct causal predictors. The ICP framework searches for sets of possible predictors that obey this invariance principle. The result is an intersection of all such sets that, with high probability, is a subset of the set of true causal predictors (29). This idea of invariance is often referred to as modularity, autonomy (30–33), or stability (33, 34). Because the methods described by Peters et al. are based on linear Gaussian models, we used a generalized extension of this method for a binary outcome (suicidal behavior). The ICP analysis proceeded through several steps:

1. For each subset of predictor variables *X*_*s*_, *Y* was regressed on *X*_*s*_ using logistic regression;
2. Residuals were calculated using model predictions;
3. The residuals were split in two based on the environment in *E* in which they were derived;
4. A two-sample *t*-test was used to determine whether the mean of the residuals was identical for each environment;
5. If the *p*-value was lower than a significance level α, the subset was accepted;
6. Once all subsets were tested, the estimated causal predictors were defined as the intersection of all accepted subsets.

While the method proposed by Peters et al. (12) uses a combination the *t*-test and the *F*-test, it suffices in our case to perform only the *t*-test because of its relation to the the F statistic. Because the number of possible subsets increases exponentially with the number of predictors, conducting ICP on many predictor variables quickly becomes computationally difficult. To overcome this barrier, lasso (35) methods can be used to select the variables that are most likely to be causal predictors. Subsets are more likely to be accepted as the significance level drops. Consequently, inferred invariant predictors must be in a larger number of accepted subsets. Multiple significance levels (i.e., α = 0.1, 0.05, and 0.01) were therefore considered to examine outcomes under increasingly strict assumptions.

## Results

Descriptive statistics and mean clinical scores at baseline are summarized in Table 1. Fifty-eight (2.1%) of participants reported suicidal behavior during the one-year follow-up. Results from ICP analyses are summarized in Table 2. When all predictor variables were used, shame demonstrated invariance at α < 0.1 across two environments: prior NSSI and age. No variable sets were invariant across any environment at the more stringent α < 0.05 and α < 0.01 levels. When using the lasso method, multiple sets demonstrated invariance at α < 0.1 across three environments (prior SI, race, and age) and one set demonstrated invariance at α < 0.05 across one environment (prior NSSI). No variable sets were invariant across any environment at the most stringent at α < 0.01 level. Shame and NA were contained in three invariant sets, the SCS-R was contained in two invariant sets, and internal entrapment was contained in one invariant set.

**Table 1:** Sample demographics and clinical descriptors.

**Variable**	***n* (%)**	
**Gender**		
Male	1,380 (51.3)	
Female	1,279 (47.5)	
Other	9 (0.3)	
Prefer not to Answer	17 (0.6)	
Unknown / Missing	59 (2.2)	
**Race**		
White / Caucasian	1,811 (67.3)	
Black / African American	506 (18.8)	
Asian	115 (4.3)	
Native Amer. / Alaska Native	123 (4.6)	
Pac. Isl. / Native Hawaiian	44 (1.6)	
Other	272 (10.1)	
**Hispanic / Latino Ethnicity**		
Yes	415 (15.4)	
No	2,199 (81.7)	
Other	20 (0.7)	
Prefer not to answer	51 (1.9)	
Prior suicidal ideation	774 (28.8)	
Prior suicide attempt	238 (8.8)	
Prior non-suicidal self-injury	312 (11.6)	
	M (SD)	Range
Age	40.4 (19.6)	18–85
DES-IV guilt	6.8 (3.4)	3–15
DES-IV shame	6.4 (3.4)	3–15
DES-IV inward hostility	5.2 (3.1)	3–15
ES internal	5.8 (8.8)	0–40
ES external	3.3 (5.7)	0–24
ISEL appraisal	12.8 (2.9)	4–16
ISEL belonging	12.3 (3.0)	4–16
ISEL tangible	12.4 (2.7)	4–16
I-PANAS-SF positive affect	13.1 (5.0)	5–25
I-PANAS-SF negative affect	7.9 (3.9)	5–25
PC-PTSD	1.1 (1.5)	0–4
PHQ-9	6.0 (6.0)	0–27
PHQ-9 suicide ideation item	0.1 (0.5)	0–3
SCS-R	6.6 (11.0)	0–64
ACSS-FAD	14.2 (4.0)	0–32
BRFLI	53.0 (16.6)	14–84
ISI	16.4 (7.2)	7–35

DES-IV, Differential Emotions Scale-IV; ES, Entrapment Scale; ISEL, Interpersonal Support Evaluation List; I-PANAS-SF, International Positive and Negative Affect Scale-Short Form; PC-PTSD, Primary Care Posttraumatic Stress Disorder Scale; PHQ-9, Patient Health Questionnaire-9; SCS-R, Suicide Cognitions Scale-Revised; ACSS-FAD, Acquired Capability for Suicide Scale-Fearlessness About Death; BRFLI, Brief Reasons for Living Inventory; ISI, Insomnia Severity Index.

**Table 2:** Results of ICP models predicting occurrence of suicidal behavior during 1-year follow-up.

**Method**	**Environment**	**α <0.1**	**α <0.05**	**α <0.01**
Full				
	Prior SI	–	–	–
	Prior SA	–	–	–
	Prior NSSI	Shame	–	–
	Race	–	–	–
	Age	Shame	–	–
	Gender	–	–	–
Lasso				
	Prior SI	SCS-R	–	–
	Prior SA	–	–	–
	Prior NSSI	None	Shame, NA	–
	Race	Shame, ES (int), NA	–	–
	Age	Shame, NA, SCS-R	–	–
	Gender	–	–	–

SI, suicidal ideation; SA, suicide attempt; NSSI, nonsuicidal self-injury; SCS-R, Suicide Cognitions Scale-Revised; ES (int), Entrapment Scale internal subscale; NA, negative affect.

## Discussion

To our knowledge, this is the first study to use the invariance-based causal prediction (ICP) method to identify key mechanisms of action underlying the emergency of suicidal behavior. Our results yielded some consistencies. In both our full and lasso models, shame demonstrated invariance across two environments—prior NSSI and age—indicating the relationship of shame with suicidal behavior remained changed minimally across patients with and without prior NSSI, and across patients ≤ 23 years and patients 24 years and older. In our lasso model, shame also demonstrated invariance across White and non-White participants. Thus, of the many predictor variables tested in this study, shame was most likely to be directly causal. This conclusion is tempered, however, by the fact that invariance was most likely to be observed when α < 0.1 and when using a reduced set of predictor variables (i.e., the lasso method). In other words, invariance was primarily observed when the analytic conditions were less stringent. Shame also did not demonstrate invariance in several especially key environments—prior suicidal ideation, prior suicide attempt, and gender—suggesting the relationship of shame with later suicidal behavior changed across these conditions. The existence of shame, or any other predictor, as a direct causal predictor is further complicated when accounting for the strict assumptions that enable the identifiability of direct causal predictors in ICP. Such assumptions are likely not met in most real-world applications.

In this study, no single variable or set of variables were consistently identified across environments. This pattern is consistent with the indeterminacy hypothesis, which posits that suicide is best understood as a complex (vs. simple or complicated) phenomenon (11). As defined by Huang et al. (10), *simple* phenomena are caused by one or a small number of factors that are both necessary and sufficient, *complicated* phenomena are caused by a large number of factors that are both necessary and sufficient, and *complex* phenomena are caused by many (but not necessarily all) combinations of factors, none of which are necessary.

Our results should be considered in light of several limitations. First, only 2.1% of participants reported suicidal behavior during follow-up. This rate is consistent with estimated annual prevalence rates of non-fatal suicidal behavior (36) but creates significant imbalance that increases the difficulty of correctly predicting the smaller group. In fact, it is common for models to correctly predict only the larger group. Such a bias may be preventing the identification of more consistent causal variables across environments. Consequently, several alternative non-linear ICP approaches could be better suited for this task (29). Many of these approaches focus on different conditional independence tests to assert invariance. In this analysis, the approaches proposed in Heinze-Deml et al. (29) did not provide any significant conclusions. Thus, we do not include them in the results. We also considered using the conditional permutation test (37) and the conditional randomization test (38), but decided against these options because these methods require a close to exact knowledge of the model, which was not available in this study. We also considered an invariance approach that is measured using variance between conditional entropies. There were three main concerns with this approach, however: (1) the risk factors considered in this sample were not discrete enough to accurately estimate conditional entropy, (2) choosing the threshold parameter was unclear, and (3) excessive dimensionality, which degrades the quality of the conditional estimates as the number of risk factors grows.

Our study is also limited by our use of self-report methods, which may be vulnerable to motivated responding and measurement error. As has been discussed elsewhere, assessing constructs defined primarily using verbal methods (e.g., surveys, questionnaires) can lack precision and frequently share a significant amount of variance with other similar variables. Millner et al. (39), for example, have argued that self-report measures can have low precision because items can be interpreted in different ways. Although self-report methods can be limited, they are widely used in clinical and research settings to assess relevant risk and protective factors. Finally, because data were collected only from beneficiaries eligible for services from the military healthcare system, our results may not generalize to other populations like civilians and veterans. Findings also may not generalize to populations at different development stages (e.g., youths or older adults) and/or non-medical settings (e.g., schools, communities). Further research using the ICP method with data collected with other assessment methods (e.g., biobehavioral tasks, interviews) and from additional populations are warranted to determine if similar results are achieved under those conditions.

Overall, our results lend further support for the perspective of suicide as a complex phenomenon that is not caused by any single psychological variable or set of variables. The inability to identify any set of psychological variables that consistently predict suicidal behavior across conditions highlights the need for multiple prevention strategies that target different combinations of risk and protective factors as well as non-psychological variables like access to potentially lethal suicide attempt methods. Results further implicate the need for additional conceptual work to better understand and describe the psychological processes underlying the emergence of suicidal behavior.

## Data availability statement

The raw data supporting the conclusions of this article will be made available by the authors upon reasonable request. Requests to access the datasets should be directed to CB.

## Ethics statement

The studies involving human participants were reviewed and approved by Naval Health Research Center. The patients/participants provided their written informed consent to participate in this study.

## Author contributions

Concept and design: AG and YX. Obtained funding, data acquisition, administrative, technical, material support, and supervision of data collection: CB. Data analysis: AG and YX. Data interpretation and drafting of manuscript: AG, YX, and CB. All authors contributed to the article and approved the submitted version.

## Funding

This project was supported by the Office of the Assistant Secretary of Defense for Health Affairs, through the Defense Medical Research and Development Program under Award No. W81XWH-14-1-0272 (PI: CB). The views expressed in this article are those of the authors and do not necessarily reflect the official policy or position of the Department of the Navy, Department of Defense, nor the U.S. Government.

## Conflict of interest

Author CB reports grant funding from the Department of Defense. The remaining authors declare that the research was conducted in the absence of any commercial or financial relationships that could be construed as a potential conflict of interest.

## Publisher's note

All claims expressed in this article are solely those of the authors and do not necessarily represent those of their affiliated organizations, or those of the publisher, the editors and the reviewers. Any product that may be evaluated in this article, or claim that may be made by its manufacturer, is not guaranteed or endorsed by the publisher.
